# Active Role of the Necrotic Zone in Desensitization of Hypoxic Macrophages and Regulation of CSC-Fate: A hypothesis

**DOI:** 10.3389/fonc.2018.00235

**Published:** 2018-06-25

**Authors:** Maryam Mehrabi, Fatemeh Amini, Shima Mehrabi

**Affiliations:** ^1^Independent Researcher, Newcastle, United Kingdom; ^2^Institute of Neuroscience and Psychology, University of Glasgow, Glasgow, United Kingdom; ^3^Internal Medicine, Iran University of Medical Sciences, Tehran, Iran

**Keywords:** necrotic zone, solid tumors, tumor-associated macrophages, macropinocytosis, cancer stem cells, anaerobic metabolism

## Abstract

Fast-proliferating cancer cells in the hypoxic region face a shortage of oxygen and nutrients, undergo necrotic cell death, and release numerous signaling components. Hypoxia-induced chemo-attractants signal for macrophages/monocytes to clear debris and return the system to steady state. Accordingly, macrophages arrange into pre-necrotic positions, where they are continuously exposed to stress signals. It can thus be hypothesized that gradual alteration of gene expression in macrophages eventually turns off their phagocytic machinery. Uncleared cell corpses within the hypoxic region potentially provide a rich source of building blocks for anaerobic metabolism of cancer stem cells *via* macropinocytosis, and are conceivably implicated in tumor progression and invasion.

## Introduction

Chaotic cell death in the necrotic zone of solid tumors, occurring as a constituent of the stress response, results in the release of cytoplasmic cell contents (1, 2). The existence of such an area within the tumor evidences the inefficient clearance of cell debris by macrophages. Macrophages/monocytes, which are recruited to this area following hypoxic induction of chemo-attractants and trails of necrotic debris, immobilize between non-necrotic and pre-necrotic zones (3). Following integration of stress signals in this microenvironment, macrophages undergo chromatin changes, such that the expression level of receptors safeguards tissue turn over and homeostasis (2, 4). As professional phagocytes, they have the opportunity to use any distinct mechanisms or a combination of receptors/co-receptors and bridging molecules to carry out this task, an example being the upregulation of “eat me” signals (5, 6). This process is not without end, however, and phagocytes most likely become desensitized after a few cycles of clearing debris due to activation of mechanisms, such as negative regulatory feedback loops (7). Following redundancy of this macrophage cohort, new blood monocytes are recruited by tumor tissue to deal with the task. This cycle continues and increasing numbers of monocytes recruited for clearing cell debris in this zone settle here and become redundant (8). Thus, it is a matter of course that an increased number of macrophages within the pre-necrotic zone is directly correlated with an elevated number of necrotic cell deaths and grade of carcinoma (9). Histopathology samples from patients of various carcinomas support this concept (10).

Recent clinical trials using blockers of immune checkpoints have been successful in improving patient conditions (11). This treatment strategy can be improved further by including molecules involved in the equation of clearing dead cells (12, 13). However, there are critical questions that need to be answered, one of which regards the role and function of macrophages located in different topological positions of solid tumors: namely the pre-necrotic zone, surrounding blood vessels, and within the stroma microenvironment (3). Our assumption is that desensitization of macrophages, which are overwhelmed with a variety of stress signals and an enormous amount of cell debris within the necrotic zone, leaves the region uncleared. In this unfavorable condition, tumor cells adopt survival metabolic pathways and constitutively scavenge available building blocks from the extracellular environment (14). This is a point of crisis, providing necessary nutrients for the uncontrolled proliferation of cancer stem cells (CSCs), dispatching exosomes, cytokines, and other factors for paracrine activities, and generating the potential to make the tumor invasive. We propose a clinical benefit of interrupting the formation of such a microenvironment, or equally preventing the generation of a similar region during cancer treatments such as radiotherapy and chemotherapy (13).

## Development of Carcinomas

Carcinomas account for 90% of all human cancers and originate within the epithelium. Formation of carcinoma initiates with abnormal proliferation of a single or small group of mutated cells, followed by selection for rapidly growing populations. Cells become malignant in a multistep process incorporating a progressive series of alterations. Many internal or environmental factors may be involved in this development, including radiation and chemical carcinogens, which initiate the process by inducing DNA damage, or phorbol ester, which stimulates cell proliferation through activation of protein kinase C. Various human cancers, such as liver and cervical carcinomas, are induced by viruses (15).

Evolution of the tumor mass leads to formation of an organ-like structure with a multilayer epithelium, various cell types, and an extracellular matrix. Distribution of these components and their complex interactions often resemble those associated with developing organs; this organization assists pathologists to classify the stage of malignancy (16). Phenotypic modifications of these cells occur under the influence of numerous factors within the tumor microenvironment (TME), such as IL-4, IL-13, TGFβ, and IL-10. Tumor-associated macrophages (TAMs) do not fit in any rigid classification of macrophages, but for the most part resemble the M2 class (17). These cells express a series of markers, including CD163, the Fc fragment of IgG, C-type lectin domains, and heat shock proteins (18–20), and secrete mitogenic factors for neoplastic cells which potentiate tumor growth, promote angiogenesis, and enable metastatic spread (21). TAMs stimulate tumor distribution through interaction with the receptor activator of the NF-κB ligand (EGF or RANKL) secreted by tumor cells (22).

## Hypoxia and the Necrotic Zone

Solid tumors are highly heterogeneous, and often exhibit low oxygen tension which increases with size (23). Cell outgrowth and shortage of blood vessels results in the formation of hypoxic regions; specifically, 0.08 mmHg O_2_ in tumors larger than 2 cm^3^ compared to 66 mmHg in normal tissue (24). Available oxygen within the tumor mass is consumed by cells close to vasculature, meaning that distant, proliferating cells face a lack of oxygen and nutrients; consequently, most of them undergo cell death and generate a necrotic zone. Cells that are clonally selected by this microenvironment are resistant to hypoxia, cell death, and therapeutic methods (25), and form the most invasive CSCs (26, 27).

**Figure 1:**
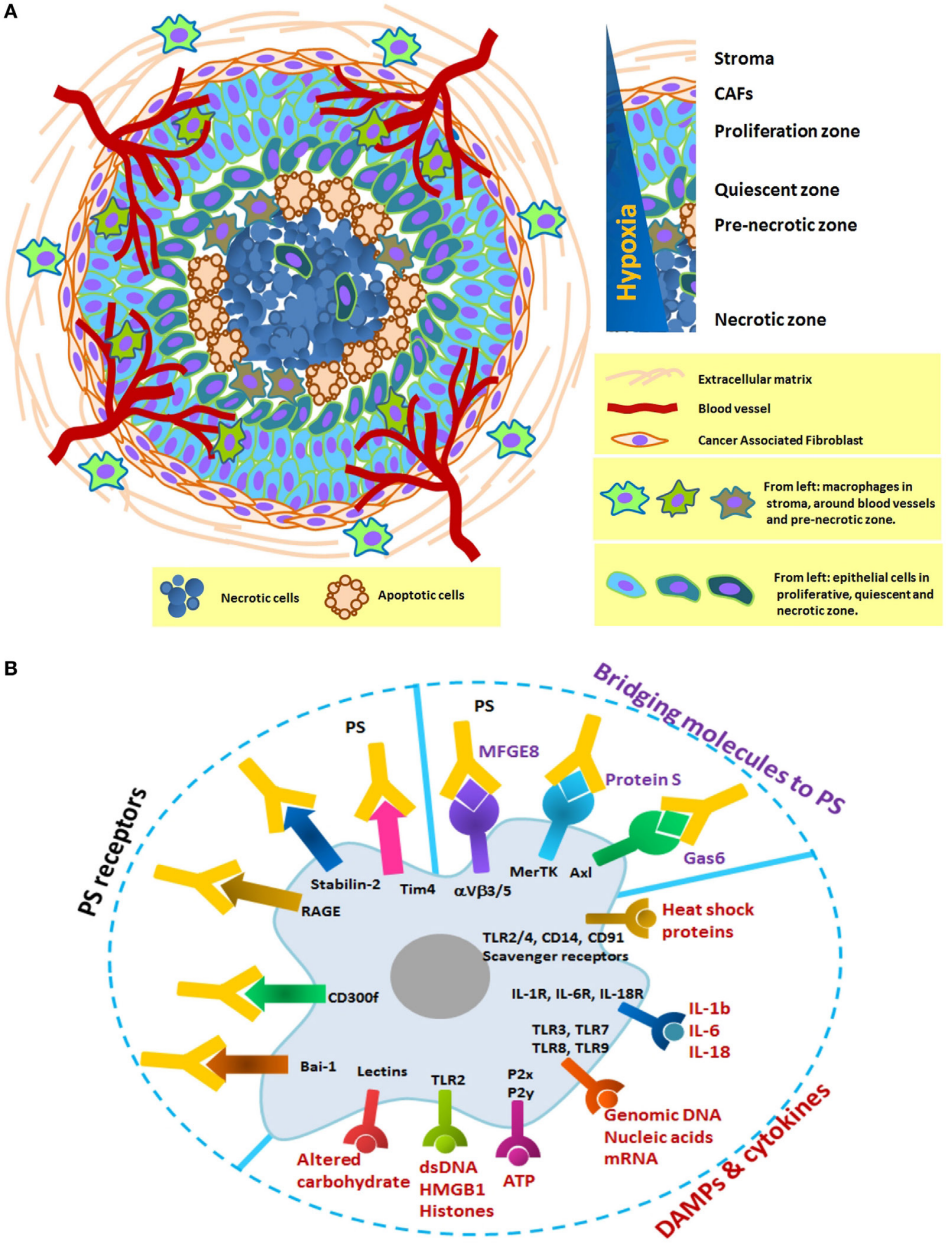
**(A)** Schematic formation of a distinct necrotic zone in carcinoma and immobilization of macrophages in three distinct areas: the stroma, the vicinity of blood vessels, and the pre-necrotic zone. Tumor macrophages originate from tissue-resident cells or blood monocytes and adapt to perform a specific function depends on their local microenvironment. In hypoxia, they progressively change from M1 to M2-like phenotype with poor antigen presentation and increase in number as tumor grows. Their receptors in this zone are continuously engaged with necrotic debris and apoptotic cells. There is, therefore, a definite need to look at the prolonged changes in the phagocytic machinery of macrophages. **(B)** Selected macrophage receptors that potentially mediate recognition of damage-associated molecular patterns (DAMPs), cytokines, and PS in the necrotic zone. DAMPs and cytokines can activate macrophages through the multiple surface receptors. Exposed PS on the surface of necrotic debris or apoptotic cells can be recognized directly by PS receptors (Bai-1, Tim4, etc.) or indirectly by bridging molecules (Gas6, protein S, and MFGE8).

Data from *in vivo* models of carcinoma and 3D spheroid cultures strongly suggest that necrotic cell death occurs at a strikingly constant distance from blood vessels (28–30). Development of cell death due to diffusion gradients follows a progressive course across three separate zones: proliferation at the outer zone, differentiation in the middle zone, and eventual, central cell death (Figure 1A). The central zone, which develops to a predictable radius of 300–400 µm, is initially necrotic, and apoptosis occurs as the spheroidal diameter increases (31).

The topography of necrosis is a major determinant of the rate of cell proliferation to angiogenesis, and is an independent prognostic factor for patients with renal, lung, thyroid, and colorectal carcinoma. A substantial proportion of necrosis in histopathology samples have been proposed as indicators of tumor aggressiveness, which generally leads to a poor clinical outcome (32–35). Clinical data show that TAMs immobilize between transient (avascular and non-necrotic) and pre-necrotic areas of human breast tumors (36, 37), and prostate (38), endometrial (39), ovarian (40), and lung carcinomas (41).

## Hypoxic TME Defines CSC-FATE

Emerging evidence suggests that hypoxic TME can potentially regulate cell fate and enrich the stem cell phenotype of cancer cells (42–44). Tumor cells located in the hypoxic region of clinical samples express stem cell-associated genes and show strong nuclear accumulation of hypoxia-inducible factor-1α (HIF-1α) protein (45–47). Previous research has established that HIF proteins activate-specific signaling pathways, such as Notch, as well as the expression of transcription factors such as Oct4 that dictate multipotency and stem cell self-renewal. Additionally, oxygen-independent oncogenic signaling pathways, such as PI3K/Akt, IGF2/IGF1R, and TGFα/EGFR, can stabilize HIF proteins (48). In a systematic literature review, Keith and Simon suggest that “hypoxic tumor tissues could be a breeding ground for cancer stem cells” (49).

## Clearance of the Necrotic Zone

Clearance of dead cells and debris is an important regulatory mechanism that has been conserved throughout evolution to serve the regulation of normal tissue homeostasis. The turnover rate of removing dead cells has been estimated to be one million cells per second (50). This process is extremely high capacity and efficient, such that dead cells are rarely seen in healthy individuals. Several steps are involved in prompt cell clearance, including recruitment of macrophages, sensing apoptotic and necrotic cells *via* “find me” signals, recognition *via* “eat me” signals, the signaling pathways that regulate cytoskeletal rearrangements necessary for engulfment, and the immune response of phagocytes in the clearance event.

Development of the necrotic zone in carcinomas is an ongoing, dynamic process. Necrotic cell death in the hypoxic microenvironment is rapid and extensive, and secondary necrosis of apoptotic cells is also common, such that macrophages cannot clear debris in a timely and efficient manner. Confusion regarding the role of necrotic cells in the development of solid tumors is widespread, as it is associated with compensatory cell proliferation and inflammation. In fact, several reports suggest that inhibition of cell death is protective against cancer development (51–55).

Necrotic corpses, apoptotic cells, and CSCs in contact with pre-necrotic macrophages can trigger engulfment *via* interaction with phagocytic receptors capable of decoding their cognate ligands. Even subtle differences in the internalized ligands could have far-reaching consequences on antigen presentation to T, B, and NK cells and become the key determinant of immunogenic or tolerogenic responses. Here, we present a conceptual overview using selected studies to propose an active role for the necrotic zone in tumor progression and invasion.

Intolerable conditions within hypoxic lesions of tumors induce chaotic breakdown of cells, resulting in infiltration of increased macrophages/monocytes. The pattern of migration is partly due to hypoxic induction of chemo-attractants, such as VEGF, CCL2, and CCL5, as well as “find-me” and danger signals along a trail of necrotic debris (56, 57). This is likely important for maximizing their opportunity to clear cell debris. Cytosolic constituents pouring into the microenvironment through the damaged membrane can interact with phagocytic receptors and initiate internalization of necrotic targets through macropinocytic mechanisms. Such eat-me signals include endogenous danger signals loaded with heat shock proteins, nuclear proteins like high-mobility group box-1 protein (HMGB1), histones, ATP, DNA, RNA, other nucleotides, and components of the extracellular matrix that are cleaved by cellular proteases (58–60). Negatively exposed phosphatidylserine (PS) is also involved in the recognition and engulfment of necrotic cells, but with a distinct and non-competitive mechanism compared to apoptotic cells (60, 61).

## How Macrophages Clear Debris

Macropinocytosis enables macrophages to continuously sample and internalize their extracellular environment at a rate of up to twice their surface area per hour. They use distinct and often unrelated receptors and bridging molecules, meaning that complete inhibition of engulfment machinery has never been achieved. This highly redundant system with various receptors mediates recognition of damage-associated molecular patterns (DAMPs), including heat shock proteins, cytokines, DNA, RNA, metabolic ATP, HMGB1, histones, and altered carbohydrates. Exposed PS on the surface of necrotic corpses is recognized directly by its receptors (Tim4, stabilin-2, RAGE, CD300f, and Bai-1) or indirectly by bridging molecules (Gas6, Protein S, and MFGE8) (Figure 1B). In general, the physiology of macrophages dramatically alters following uptake of these components, leading to modification of protein expression and cytokine production. Internalized antigens are processed and loaded onto MHC molecules for presentation to immune cells (62). Both MHC class I and II have been identified on macropinosome-like structures (63–65). The macropinocytic process is expected to provoke an inflammatory response (66). However, contradictory results from cancer patients and *in vivo* models support the complexity of the TME (60, 67).

## Potential Mechanisms in HAMS Alteration

### Chromatin Remodeling

The necrotic zone is a non-resolving inflammatory condition, and macrophages/monocytes continue to enter the zone and differentiate as the lesion progresses. DAMPs derived from necrotic cancer cells can foster this chronic condition through stimulation of TLRs and specific plasma membrane receptors in macrophages (Figure 1B). With enormous functional plasticity, HAMs integrate these signals, leading to alteration of their regulatory state and transcriptional program. For example, HMGB1 activates an immune response *via* TLR signals and regulates intracellular transcription (68).

Molecular studies of macrophages show similar trends in response to a single polarizing stimulus. Results obtained from gene expression profiling of a 20,000-element cDNA microarray in LPS-stimulated murine macrophages show major changes in expression of a broad range of genes with comparison to normal tissue (4, 69–72). Interestingly, the expression level of these genes across five-time points after LPS challenge has a profound effect on the macrophage transcriptome, and both differentiated TRMs and monocyte-derived macrophages become less plastic over time (73–76). Most of the genes initially expressed at a high level are repressed, as if to accommodate the new spectrum of induced genes. Genes which were initially undetectable are promoted, and very few elements also remain static. Every macrophage population in these experiments is likely to be different (77). A similar tendency has been reported in response to other stimuli, including a TLR2-ligand (MALP2), a TLR9-ligand (CpGs), M-CSF (78), GM-CSF (79), and exposure to cytokines, such as IL-1β, IL-4, tumor necrosis factor (TNF)α, IFNγ, or TGFβ (80). Results suggest that changes are persistent even after stress signal exposure is seized, and are faster and stronger with each consecutive exposure.

The phenotype of macrophages during hypoxia is expected to be similar to that of LPS-stimulated macrophages, which is M1-like. However, HAMs are associated with an M2-like response (81). *In vivo* tumor models of glioma and breast cancer have demonstrated macrophage migration as a function of hypoxia, and suggest evolving polarization to M2, concomitant to the hypoxic shift within the growing tumor (82, 83). Taken together, these results appear to support our assumption that continuous exposure to a broad range of stimuli determines a continuum of distinct transcriptional and functional output in HAMs. As time goes on, HAMs exhibit gradual changes in their receptor expression anywhere between M1 and M2. In fact, the engulfment machinery of macrophages is engaged with a continuous cycle of necrotic debris, leading to significant, prolonged changes in involved receptors. Disarmed macrophages are not able to recruit or present antigens to other immune cells and block the adaptive immune response. Macrophages may eventually become senescent or undergo necrotic cell death, thus joining the crew of the necrotic zone.

### The Hypoxic Response

Macrophages respond to hypoxia through activation of the transcription factor, hypoxia-inducible factor-1, which is an oxygen sensor and plays a significant role in macrophage polarization (84). This master transcription factor is regulated by nuclear factor-κB (NF-κB) and induces profound changes in the expression level of angiogenesis- and metastasis-related genes, such as VEGF, FGF2, MMP7, and MMP9 (38, 52, 53, 85). Consequent to this is recruitment of more macrophages, and release of pro-inflammatory cytokines, such as TNFα, IL-1β, MIF, CCL3, and COX2, as well as M2 markers, such as IL-10 and arginase 1. A clear link between the HIF responses of innate immunity and cellular processes that aid the engulfment processes in macrophages has been established in previous research (86, 87). The continued upregulation of HIF is part of the regenerative and immunosuppressive response (88).

### Negative Regulatory Feedback Loops

Here, we argue that continuous inflammatory events within the necrotic zone cause prolonged changes in macrophage phagocytic receptors. A well-known example is aberrant expression of TAM-receptors (Tyro, Axl, Mer) during tumor progression in many cancers (89).

Binding of inflammatory cytokines to TLRs and costimulatory receptors in HAMs can result in activation of signaling cascades, causing upregulation of TAM-receptors. The innate immune response is a carefully regulated system, meaning that unrestrained signaling by TLRs and cytokine receptors is not supported. A notable mechanism to inhibit these immune responses in APCs is the negative regulatory pathway driven by the TAM-receptor tyrosine kinases. These receptors bind their cognate ligand using bridging molecules, like Gas6 or protein S, which sit between them and PS on the surface of apoptotic cells, necrotic debris, and CSCs. The suppressor of cytokine signaling proteins (SOCS1 and SOCS3) are among the most important genes that are induced by this negative feedback loop. TAM-receptor signaling also inhibits TLR3, TLR4, and TLR9 activation and their multiple points in signal transduction cascades, including the activation of the p38 mitogen-activated protein kinase, extracellular-signal-regulated kinase 1 (ERK1/ERK2), NF-κB, and TNF-receptor-associated factor (TRAF3 and TRAF6). TLR-induced production of proinflammatory cytokines, including TNFα, interleukins (IL-6, IL-12), and type 1 interferons (IFNs), are also inhibited as result of this signaling (90). Our assumption is that this feedback loop is likely to desensitize macrophage receptors in the hypoxic area and reduce their rate of turn over and phagocytic uptake.

### Immune Checkpoints

The reduced phagocytic capacity of HAMs could also be a consequence of signaling pathways triggered by immune checkpoints. Increased expression in tumor cells of such signals, like CD47 and programmed death-ligand 1 (PD-L1), is proposed to be a mechanism through which cancer cells induce “don’t eat me” signals and evade immune detection by T cells. Immune checkpoint blockade has been the subject of multiple clinical trials in cancer, but has tended to focus on the functional consequences of T cells rather than macrophages (11, 91, 92). Interestingly, a limited number of research has demonstrated an increase in phagocytosis and reduction in tumor growth in a macrophage-dependent fashion (93, 94).

Overexpression of CD47 in cancer cells activates SIRPα, which is an inhibitory receptor expressed mainly by myeloid cells. Upon binding CD47, SIRPα initiates a signaling cascade through phosphorylation of the immunoreceptor tyrosine-based inhibition motifs on its cytoplasmic tail (95). Subsequent binding and activation of SHP-1 and SHP-2 in macrophages prevents accumulation of myosin-IIA at the phagocytic synapse and blocks phagocytosis (96). Antibody blocking of this regulatory signal in normal tissue does not induce phagocytosis, although it does turn off the “don’t-eat-me” signal; this is owing to the fact that in the absence of CD47/SIRPα signaling, a secondary pro-phagocytic “eat-me” signal is required to subject cells to phagocytosis (12). There are several “eat-me” signals which have the potential to trigger this process, such as surface calreticulin (CRT) and PS (97, 98). Cell-surface CRT is present on a subset of all solid tumors, with no expression on normal cells (99). CRT interacts with CD91 on macrophages and is required for phagocytosis of tumor cells following neutralization of the CD47/SIRPα interaction (100). It has been reported that cell-surface expression of CRT is controlled by the TLR-Btk pathway, and that a TLR4 agonist is necessary for activation of macrophages after blocking of the CD47/SIRPα interaction (101).

Another clinically successful immune checkpoint, the PD-1 receptor, is primarily known for its role in the inhibition of stimulated T cells (102). Its phagocytic effect, however, has recently been considered. TAMs in humans express variable levels of PD-1 which increase with tumor progression in the M2 subset, and their upregulation over time correlates negatively with phagocytic potency against tumor cells in human cancer. PD-L1 removal in animal models rescues PD-1^+^ phagocytosis in macrophages and decreases tumor size. The M2 subset of TAMs mainly originates from bone marrow and resides in the inflammatory TME (103).

### Final Thoughts

**Figure 2:**
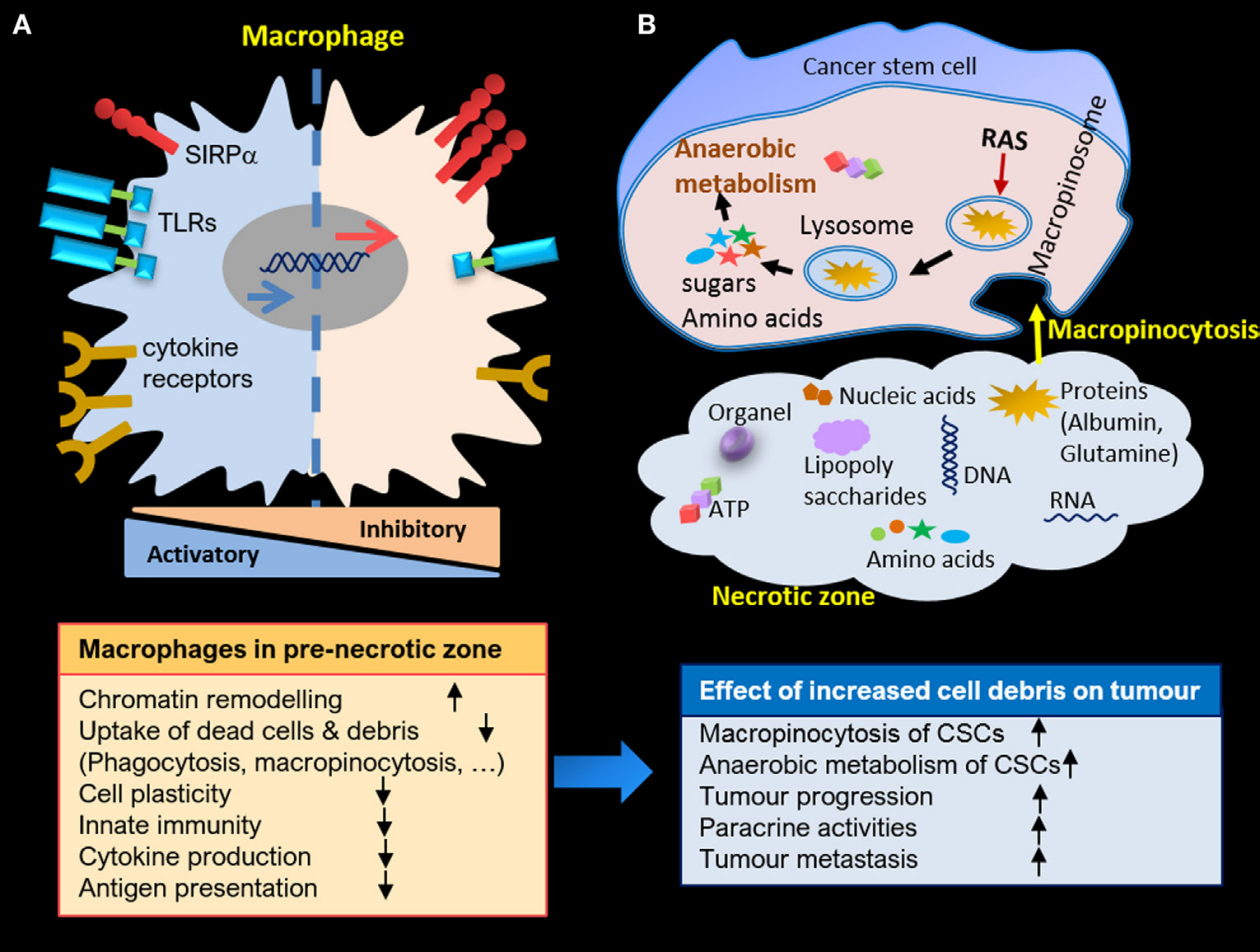
**(A)** Macrophage alteration in pre-necrotic zone and gradual loss of activities due to continuous exposure to cell debris. **(B)** Anaerobic metabolism of cancer stem cells (CSCs) in necrotic zone through macropinocytosis, and enhanced proliferation and paracrine activities of CSCs due to accumulation of cell debris in necrotic zone.

Here, we have focused on the role of HAMs in the clearance of cell debris resulting from stress stimuli in the necrotic zone of solid tumors. The aim has been to highlight how the engulfment machinery of macrophages may alter due to interaction with endogenous danger signals. This alteration may worsen over time because of continuous exposure to presented stimuli. Therefore, the uncleared necrotic zone is perhaps the most strategic region in carcinoma, an area in which CSCs can ingest essential building blocks for their survival and maintain anaerobic metabolism in the hypoxic microenvironment. Consequently, cells growing within this harsh milieu are highly aggressive and capable of developing drug and therapeutic resistance. Further proliferation of CSCs jointly with necrotic corpses could tear the carcinoma capsule, resulting in their deposition in blood vessels, lymph nodes, and ultimately resulting in metastasis (Figure 2).

The key to avoiding these processes, therefore, is to clear necrotic debris in the initial stages, with the implication that engulfment machinery of HAMs should be up and running with no alteration. Treatment of HAMs with any agent, however, will not be the answer, as they are under the continuous influence of necrotic debris. A potential therapeutic target would be the genetic modification of macrophages for key engulfment receptors. Gene delivery vehicles could also be transported directly to on-site macrophages. We speculate that this strategy may partly preserve both the engulfment capacity and the immunogenicity of macrophages against CSCs, as phagocytosis is the gatekeeper for immune responses against danger signals.

Another implication of this hypothesis would be to avoid inducing further hypoxic cell death during therapeutic courses. Considering this strategy in conjunction with imaging technologies and computational modeling of the TME would allow surgeons to choose a better approach to eradicate a mass of solid tumor.

## Notations

Macrophages refer to tissue-resident population, TAMs: tumor-associated macrophages (TAMs) regardless of their position, and hypoxic-TAMs (HAMs) denotes TAMs allocated in the pre-necrotic zone of solid tumor, TAM-receptors: Tyro, Axl, Mer.

## Author Contributions

MM: conception and design of manuscript, research of supporting evidence, writing and design of figures. FA and SM: editing, critical revision of content and final approval.

## Conflict of Interest Statement

The authors declare that the research was conducted in the absence of any commercial or financial relationships that could be construed as a potential conflict of interest.
